# Antenatal pelvic floor muscle training and urinary incontinence: a randomized controlled 7-year follow-up study

**DOI:** 10.1007/s00192-021-05028-x

**Published:** 2021-12-22

**Authors:** Signe Nilssen Stafne, Rebecka Dalbye, Oda M. Kristiansen, Yvonne E. Hjelle, Kjell Åsmund Salvesen, Siv Mørkved, Hege Hølmo Johannessen

**Affiliations:** 1 Department of Public Health and Nursing, Norwegian University of Science and Technology, P.O. Box 8905, 7491 Trondheim, Norway; 2 Department of Clinical Services, St. Olavs hospital, Trondheim University Hospital, Trondheim, Norway; 3 Department of Nursing and Health Promotion, Oslo Metropolitan University, Oslo, Norway; 4 Department of Obstetrics and Gynecology, St Olavs Hospital, Trondheim University Hospital, Trondheim, Norway; 5 Department of Clinical and Molecular Medicine, Norwegian University of Science and Technology, Trondheim, Norway; 6 Department of Health and Welfare, Østfold University College, Fredrikstad, Norway; 7 Department of Physical Medicine and Rehabilitation, Østfold Hospital Trust, Sarpsborg, Norway

**Keywords:** Long-term effect, Pelvic floor muscle training, Pregnancy, Prevention, Urinary incontinence, Women

## Abstract

**Introduction and hypothesis:**

Urinary incontinence is common postpartum. Our aims were to assess whether antenatal exercise including pelvic floor muscle training (PFMT) has long-term effects on urinary incontinence (UI) and to explore factors associated with UI 7 years postpartum.

**Methods:**

A follow-up of a two-centre randomized controlled trial performed at St. Olavs Hospital and Stavanger University Hospital, Norway. In the original trial women were randomized to a 12-week structured exercise protocol including PFMT or standard antenatal care during pregnancy. Link to an electronic questionnaire was sent by postal mail 7 years postpartum. Prevalence of UI was assessed with Sandvik severity index and compared between groups. Factors associated with UI were studied using multivariable logistic regression analysis.

**Results:**

The response rate was 35% (298/855). UI was reported by 78 (51%) in the intervention group and 63 (57%) in the control group (*p* = 0.539). In the multivariable logistic regression analyses, women with UI at inclusion had a five-fold increase in odds of UI at 7 years (OR 5.4, 95% CI 2.6, 11.5). Engaging in regular exercise was not significantly associated with UI at 7 years; however, UI was associated with lower exercise intensity (OR 2.4, 95% CI 1.2, 4.6).

**Conclusions:**

We found no group differences of antenatal exercise including PFMT on UI after 7 years among the responders. UI in pregnancy increased the risk of long-term UI. Regular exercise was not associated with UI at 7 years; however, women with UI were more than twice as likely to exercise at lower intensity than continent women.

## Introduction

Urinary incontinence (UI) is defined as “any complaint of involuntary loss of urine” [1]. UI is the most prevalent pelvic floor dysfunction with on average 25–40% of all adult women reporting any UI [2]. Pregnancy and delivery are considered important risk factors for developing UI. During pregnancy the increased load on the pelvic floor and hormonal changes may reduce strength and function of the pelvic floor muscles (PFMs) [3]. Furthermore, nerves, connective tissue and PFM are often injured during delivery [4]. UI may reduce quality of life, and many women withdraw from social settings and physical activities because of fear of leakage and an unpleasant odour [5].

PFMs have the important role of keeping the pelvic organs in place and contribute to continence. Pelvic floor muscle training (PFMT) aims to strengthen the PFM and enhance function [6]. Targeted PFMT is effective in the prevention and treatment of UI among non-pregnant [7] and pregnant women [8]. However, only one study has examined the long-term effect of antenatal PFMT beyond the first year after delivery and found no group difference [9].

In a randomized controlled trial including 855 pregnant women, we have previously shown that including PFMT in a general antenatal exercise programme had preventive and therapeutic effects on UI in late pregnancy [10] and 3 months postpartum [11]. Since pregnancy may be a “golden opportunity” for sustainable lifestyle changes, our hypothesis was that the effect of PFMT during pregnancy may have long-term effects. In this follow-up study our aim was to explore whether an antenatal exercise programme including PFMT had long-term effects on UI and to assess possible factors associated with UI 7 years after delivery.

## Materials and methods

This was a follow-up of a two-armed, two-centre randomized controlled trial. The “Training in Pregnancy” (TRIP) study was conducted in Norway from April 2007 to June 2009 [12]. Healthy pregnant women were included in mid pregnancy (gestational week 18–22) and randomized to an intervention or control group.

The follow-up study was approved by the Regional Committee for Medical and Health Research Ethics (2014/618/REK). The original TRIP trial was approved by the Regional Committee for Medical and Health Research Ethics (REK 4.2007.81) and had been registered in www.clinicaltrials.gov (NCT00476567).

The intervention group received individual instructions on how to perform PFMT, and this was assessed by vaginal palpation at study inclusion. The intervention consisted of an exercise programme for 12 weeks and followed recommendations from ACOG [13] consisting of aerobic activity and strength training, including PFMT and relaxation exercises. The exercise programme has previously been described in detail [10, 12]. The strength training, including PFMT, followed the recommendations for effective training dosage with 8–12 repetitions and three sets to be performed 3 days per week [14]. Exercise groups led by a physiotherapist were offered 1 day a week, and women were encouraged to perform a home exercise programme twice a week. PFMT was performed in different positions with legs apart to emphasize specific strength training of the PFM and relaxation of other muscles.

Women allocated to the control group received standard antenatal care, which is free of charge and part of the public health care system in Norway. Women received written information about physical activity and PFMT, but did not receive individual instruction on PFMT.

In 2014–2016 all participants in the TRIP trial were invited to participate in a follow-up study. Women were sent postal mail including a link to an electronic questionnaire using the software CheckWare® (CheckWare AS, Trondheim, Norway). The electronic questionnaire included questions about general health, pregnancies and deliveries since inclusion, UI, PFMT, physical activity and exercise. Women with recent deliveries (< 6 months before the follow-up study), pregnant women and women who did not respond to the questions regarding UI were excluded from the present analysis.

### Outcome variable

UI was the primary outcome in the present study and was assessed using Sandvik’s severity index [15]. Women reporting no urinary leakage were categorized as urinary continent, and women reporting any symptoms of UI were categorized as incontinent according to the ICS/IUGA definition [1]. Incontinent women were further categorized as either stress urinary incontinent (SUI, loss of urine when physically active, coughing or laughing) or mixed urinary incontinent (MUI, SUI in combination with loss of urine with urgency) [1].

### Explanatory variables

Age, body mass index (BMI) and birthweight from index delivery (delivery following the intervention period in the present study) were analysed as continuous variables. Number of deliveries after inclusion were categorized as none, 1 and ≥ 2. Engaging in regular exercise, defined as weekly physical activity to maintain or improve physical fitness, was self-reported and categorized as yes/no. Furthermore, women reporting regular exercise provided information on number of days per week (1, 2, 3, 4 or ≥ 5), time used per exercise session (< 30, 30–60, ≥ 60 min) and exercise intensity (slightly, somewhat and very strenuous). For the statistical analyses exercise frequency was categorized as 1–2 days/week or ≥ 3 days/week and exercise duration as <60 or ≥ 60 min. Furthermore, exercise intensity was categorized as slightly and somewhat strenuous or very strenuous. PFMTs were categorized as never, 1 time per week and ≥ 2 times per week. Mode of index delivery was categorized as caesarean section, spontaneous vaginal delivery or instrumental vaginal delivery.

### Statistical analysis

Descriptive statistics for continuous variables are presented with mean, standard deviation (SD) and range. Categorical variables are presented as frequencies and percentages. The independent samples *t*-test was performed to compare the continuous variables such as age, BMI and birthweight, and the chi-squared test was performed to compare differences between categorical variables. To examine whether PFMT during pregnancy had long-term effects on UI, number of incontinent women was compared using chi-squared test.

We explored risk factors associated with UI 7 years after index delivery using multivariable logistic regression analyses. Variables with *p* < 0.20 in univariable analyses were included in the multivariable analysis. We applied two models in the multivariable logistic regression analyses, one included engaging in regular exercise and one included detailed information on exercise habits among those engaging in regular exercise. None of the variables in the multivariate logistic regression model were highly correlated. Effect estimates are presented as odds ratio (OR) with 95% confidence interval (CI). A significance level of 5% was used. The statistical analyses were performed using SPSS 27.

## Results

Of the 855 women participating in the TRIP trial, 298 responded (35%). Non-responders had lower level of education than responders; otherwise, no differences in baseline characteristics were found (Table 1).

**Table 1:** Maternal characteristics at inclusion stratified according to response status after 7 years (*n* = 855)

At 7 years	Responders at 7 years (*n* = 298)	Non-responders at 7 years (*n* = 557)	*P* value
Age- years	30.6(3.9)[19,44]	30.4(4.6)[20,46]	0.404
BMI kg/m^2^	24.6(3.1)[18,39]	25.0(3.3)[18,39]	0.088
Married/living with partner	295 (99.0)	539 (96.8)	0.059
Higher education (≥ 13 years at school)	277 (93.0)	483 (86.7)	**0.035**
Parity			0.457
P1	173 (58.1)	313 (56.2)	
P2	90 (30.2)	164 (29.4)	
≥ P3	35 (11.7)	80 (14.4)	
Urinary incontinent (UI) at inclusion	117 (39.3)	235 (42.2)	0.405
Birthweight (g) index delivery	3511(543)[825,4930]	3523(538)[850,4920]	0.745
Mode of index delivery			
Spontaneous vaginal delivery	227 (76.2)	418 (75.0)	0.918
Caesarean section	34 (11.4)	74 (13.3)	
Instrumental vaginal delivery	37 (12.4)	61 (11.0)	
Missing	0	4 (0.7)	

**Fig. 1:**
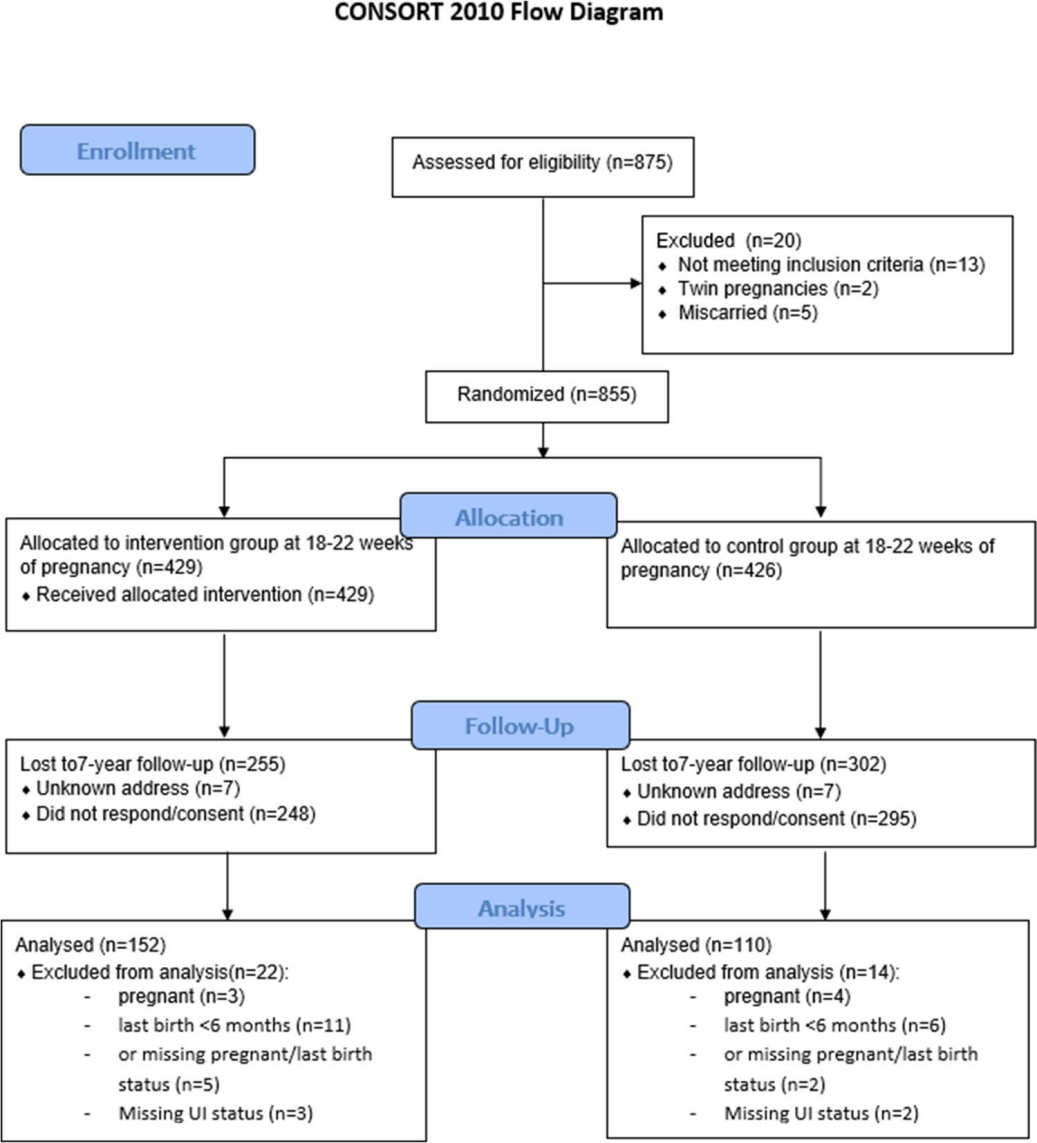
Flowchart of study participants

Women with recent deliveries (< 6 months before the follow-up study, *n* = 17), pregnant women (*n* = 7), women missing information on pregnancy or last birth status (*n* = 7) and women who did not respond to the questions regarding UI (*n* = 5) were excluded. Thus, 262 (31%) women were included in statistical analyses in the present study (Fig. 1). There were no differences in maternal characteristics between intervention and control group 7 years after inclusion. Approximately half of the women reported UI after 7 years: 78 (51%) in the intervention group and 63 (57%) in the control group (*p* = 0.539). Among the incontinent women, 58 (74%) reported SUI and 20 (26%) reported MUI in the intervention group, whereas in the control group 51 (81%) and 12 (19%) reported SUI and MUI, respectively (*p* = 0.377).

Maternal characteristics between 121 (46%) continent and 142 (54%) incontinent women were similar except for a higher proportion of women with UI at inclusion in the incontinent group (Table 2). There were no differences between continent and incontinent women regarding performing regular exercise, number of days per week and duration of exercise sessions. However, fewer incontinent women reported very strenuous exercise intensity compared to continent women (58% vs 35%, *p* = 0.002).

**Table 2:** Maternal characteristics according to the TRIP study randomisation and the study sample stratified according to UI status after 7 years (*n* = 262)

	Randomisation			UI status at 7 years		
At 7 years	Control group *n* = 110	Intervention group *n* = 152	*P* value	No UI n = 121	UI *n* = 141	*P* value
Age-years	38.6 (4.1) [27–52]	38.5 (3.9) [29–51]	0.902	38.1 (4.2) [27–52]	38.8 (3.8) [29–49]	0.138
BMI kg/m^2^	23.7 (3.8) [18–42]	23.0 (2.9) [18–33]	0.070	23.0 (3.0) [17.6–33.1]	23.5 (3.5) [17.6–41.8]	0.238
Married/living with partner	102 (92.7)	139 (91.4)	0.718	111 (91.7)	130 (92.2)	0.877
Higher education (≥ 13 years at school)	104 (94.5)	146 (96.0)	0.357	117 (96.7)	133 (94.2)	0.931
Parity			0.165			0.469
P1	6 (5.5)	18 (11.8)		12 (9.9)	12 (8.5)	
P2	56 (50.9)	79 (51.6)		66 (54.5)	69 (48.9)	
≥ P3	48 (43.6)	55 (36.2)		43 (35.5)	60 (42.6)	
Number of deliveries after inclusion			0.287			0.488
0	35 (31.8)	63 (41.4)		39 (32.3)	59 (41.5)	
1	59 (53.6)	63 (41.4)		61 (50.4)	62 (43.7)	
≥ 2	16 (14.5)	26 (17.1)		21 (17.4)	21 (14.8)	
Urinary incontinent (UI) 7 years	63 (57.3)	78 (51.3)	0.539			
Engaging in regular exercise*	73 (66.4)	106 (69.7)	0.562	77 (63.6)	102 (72.3)	0.122
Exercise frequency**			0.495			0.499
1–2 days per week	46 (63.0)	61 (57.8)		44 (57.1)	63 (61.8)	
> 2 days per week	27 (37.0)	45 (42.5)		33 (42.9)	39 (38.2)	
Exercise duration**			0.148			0.469
< 60 min per exercise session	60 (82.0)	77 (72.6)		57 (74.0)	80 (78.4)	
≥ 60 min per exercise session	13 (18.0)	29 (27.4)		20 (26.0)	22 (21.6)	
Exercise intensity**			0.504			**0.002**
Slightly strenous	2 (2.8)	1 (0.9)		0	3 (2.9)	
Somewhat strenuous	40 (55.6)	55 (51.9)		32 (42.1)	63 (61.8)	
Very strenuous	30 (41.7)	50 (47.2)		44 (57.9)	36 (35.3)	
PFMT			0.060			0.924
Never	51 (46.4)	88 (58.3)		64 (53.3)	75 (52.8)	
1×/week	54 (49.1)	48 (31.6)		42 (35)	45 (31.9)	
≥ 2×/week	5 (4.5)	15 (9.9)		14 (11.7)	21 (14.7)	
Urinary incontinent (UI) at inclusion	50 (45.5)	52 (34.4)	0.083	23 (19.0)	79 (56.0)	**< 0.001**
Birthweight (g) index delivery	3574(459)[2370–4830]	3487(604)[825–4930]	0.204	3466(552)[825–4620]	3568(542)[1290–4930]	0.114
Mode of index delivery			0.900			0.828
Spontaneous vaginal delivery	86 (78.2)	115 (75.7)		95 (78.5)	106 (75.1)	
Caesarean section	12 (10.9)	18 (11.8)		13 (10.7)	17 (12.1)	
Instrumental vaginal delivery	12 (10.9)	19 (12.4)		13 (10.7)	18 (12.8)	

*Self-reported and defined weekly physical activity to maintain or improve physical fitness

**Reported for women engaging in regular exercise (*n* = 180)

Age, regular exercise training, birthweight and UI status at inclusion showed a significance level < 0.2 in the univariable logistic regression analyses and were included in the multivariable analysis. BMI and parity were also included because of clinical relevance.

In the multivariable logistic regression model including regular exercise, UI at inclusion was the only variable significantly associated with UI after 7 years (OR 5.5; 95% CI: 3.0–10.0). (Table 3). UI at inclusion remained significantly associated with UI after 7 years in the multivariable logistic regression model exploring exercise frequency, duration and intensity. Interestingly, this model showed that women with UI were more than twice as likely to exercise at lower exercise intensity than women with no UI (OR 2.4; 95% CI: 1.2–4.6). No associations were found for exercise frequency or duration (Table 4).

**Table 3:** Associations between urinary incontinence 7 years after index delivery and age, BMI, parity, pelvic floor muscle training, regular exercise training, urinary incontinence at study inclusion, mode of delivery and birthweight. Results from univariable and multivariable logistic regression analysis (*n* = 262)

	Univariable logistic regression analyses	Multivariable logistic regression analyses
	OR (95% CI)	OR (95% CI)
Age	1.1 (1.0,1.1)	1.0 (0.9,1.0)
BMI	1.1 (0.9,1.1)	1.1 (0.9,1.1)
Parity		
1	Reference	
2	1.0 (0.5,2.5)	
≥ 3	1.4 (0.6,3.3)	
PFMT/week		
Never	Reference	
1 time per week	0.9 (0.6,1.6)	
≥ 2 times per week	1.0 (0.5,2.6)	
Engaging in regular exercise*		
No	0.7 (0.4,1.1)	0.6 (0.4,1.1)
Yes	Reference	
UI at inclusion		
No	Reference	
Yes	5.7 (3.3,9.9)**	5.5 (3.0,10.0)**
Mode of index delivery		
Normal vaginal delivery	Reference	
Instrumental delivery	1.1 (0.5,2.5)	
Caesarean section	1.1 (0.5,2.3)	
Birthweight	1.0 (1.0,1.0)	

*Self-reported and defined weekly physical activity to maintain or improve physical fitness

**p < 0.01. *OR*: odds ratio; *CI*: confidence interval; *BMI*: body mass index (kg/m^2^)

*PFMT*: pelvic floor muscle training; *UI* urinary incontinence

**Table 4:** Associations between urinary incontinence 7 years after index delivery and age, BMI, parity, pelvic floor muscle training, exercise training (frequency, duration and intensity), urinary incontinence at study inclusion, mode of delivery and birthweight. Results from univariable and multivariable logistic regression analysis (*n* = 179)

	Univariable logistic regression analyses	Multivariable logistic regression analyses
	OR (95% CI)	OR (95% CI)
Age	1.1 (1.0,1.1)	1.0 (0.9,1.1)
BMI	1.1 (1.0,1.1)	1.0 (0.9,1.1)
Parity		
1	Reference	
2	1.2 (0.5,2.8)	
≥ 3	1.5 (0.6,3.5)	
PFMT/week		
Never	Reference	
1 time per week	0.9 (0.6,1.5)	
≥ 2 times per week	1.0 (0.4,2.4)	
Exercise frequency		
1–2 days/week	1.2 (0.7,2.2)	
≥ 3 days/week	Reference	
Exercise duration		
< 60 min per exercise session	1.3 (0.6,2.6)	
≥ 60 min per exercise session	Reference	
Exercise intensity		
Slightly/somewhat strenuous	2.5 (1.4,4.6)*	2.4 (1.2,4.6)**
Very strenuous	Reference	
UI at inclusion		
No	Reference	
Yes	5.9 (3.4,10.1)*	5.4 (2.6,11.5)*
Mode of index delivery		
Normal vaginal delivery	Reference	
Caesarean section	1.2 (0.6,2.7)	
Operative delivery	1.2 (0.5,2.5)	
Birthweight	1.0 (1.0,1.0)	

*p < 0.01; **p < 0.05. *OR*: odds ratio; *CI*: confidence interval; *BMI*: body mass index (kg/m^2^)

*PFMT*: pelvic floor muscle training; *UI* urinary incontinence

## Discussion

There were no group differences regarding UI after 7 years among the third of the original study population participating in the follow-up. In multivariable analyses we found that UI in mid-pregnancy was associated with reporting UI 7 years later. Furthermore, regular exercise was not associated with UI. However, when exploring exercise habits in more detail, women with UI engaging in regular exercise were more than twice as likely to exercise at lower exercise intensity.

As our short-term results show that including PFMT in an antenatal exercise programme reduced UI in late pregnancy [10] and 3 months postpartum [11], we intended to assess the long-term effects of the intervention. No group differences after 7 years are in concurrence with the only study assessing the effect of antenatal PFMT beyond 6 months postpartum [9]. However, due to the low response rate (35%) only 31% of the women attending the original trial were included in the analyses in this follow-up study. This is a major concern, and we cannot conclude on the long-term effects. Nevertheless, our results can be implemented in meta-analyses and contribute to new knowledge, as suggested in the most recent Cochrane review [8]. The value of evaluating the long-term effect of antenatal PFMT beyond 1 year after delivery has been questioned because many women will have subsequent pregnancies and deliveries. Furthermore, long-term effect of exercise may not be expected if PFMT is not maintained over time.

Continuing exercise is vital to maintain any training effect. However, the minimal level of PFMT intensity, volume and frequency needed to maintain the effect of training is not known [16]. In this 7-year follow-up study, 47% of the women reported performing PFMT regularly 7 years after index delivery, with no difference between randomized groups.

In the original TRIP study, only women allocated to the intervention group were assessed with vaginal palpation and instructed in correct PFMT at baseline. However, at follow-up in late pregnancy and 3 months postpartum all women were offered this assessment and received verbal and written information about correct PFMT. Thorough instruction in correct PFM contraction has been demonstrated to be a key factor for benefit of PFMT [8]. Some suggest that effective PFMT requires both physical and behavioural changes and that an increased focus on the cognitive and behavioural perspectives may result in a more effective implementation of and adherence to PFMT in the long term [17]. It is possible that women in the control group changed their behaviour regarding PFMT following this attention from study personnel. This may potentially have diluted the effects of the intervention.

In multivariable analyses we found that women who experienced UI during pregnancy had a six-fold risk of UI 7 years later. Others have also reported antenatal and/or postnatal UI to be associated with increased risk of UI in the long term [18–20]. Risk factors, such as BMI, age, parity, birthweight or mode of index delivery, were not associated with experiencing UI in our study. Only 22% of the women in this study were overweight (BMI ≥ 25 kg/m^2^) compared to 34% of the general female Norwegian population aged 25–44 years in 2015 [21]. This may indicate that our study population was healthier than the general female population in Norway.

One interesting finding was that performing regular exercise was not associated with UI. Although there is strong evidence that regular exercise is beneficial for a wide range of diseases and conditions, it is questioned whether the positive effects of physical activity and exercise apply to the pelvic floor [22]. Cross-sectional studies indicate that high-impact sports can be harmful, whereas low-impact sports can be protective against UI [2]. Engaging in regular exercise can hypothetically overload, stretch and weaken the PFM because of increased intraabdominal pressure [22]. In the present study, almost three in four incontinent women and three in five continent women reported regular exercise. When addressing exercise intensity, frequency and duration as explanatory variables, we found that only exercise intensity was associated with UI. Incontinent women who exercised regularly were more than twice as likely to exercise at lower intensity levels. Evaluating associations between exercise and UI is complex. Evidence from longitudinal studies suggest that exercise has a protective effect against incident UI, probably mediated through an effect on weight and BMI [2]. Most women in the present study had normal BMI, and this may have influenced our findings. In the present study we were unable to explore causality between UI and regular exercise. The association between exercise habits in detail and pelvic floor disorders in general, and incontinence in particular, should be a priority in future studies among females of reproductive age.

More than half reported UI in the present study. The responding women were on average 38 (27–52) years old and predominantly premenopausal. UI prevalence increases after menopause, and prevalence estimates in the adult female population vary from 5% to 69% [2] depending on study populations and methodological qualities. In the present study women were categorized as incontinent if reporting any involuntary leakage of UI, irrespective of frequency and severity. This is in accordance with standard terminology [1] and may explain the high prevalence. Another factor contributing to the high UI prevalence is that women with UI may have more interest in participating in a follow-up study compared to continent women.

Strengths of this study are the study design and exclusion of pregnant women or those with recent deliveries. Furthermore, identical validated outcome measures were used at all time points from inclusion to follow-up and adherence to PFMT regimens in both randomized groups was accounted for. One limitation was a low response rate of 35%. This limits the strength of the original design (RCT) and introduces the risk of type II error. Furthermore, most women (63%) had subsequent pregnancies and deliveries between the original TRIP trial and the 7-year follow-up, and we have limited obstetric data from these deliveries. Moreover, we do not have any information about PFMT performed after the index delivery in the TRIP trial. Furthermore, other health issues potentially affecting continence status may not be fully covered by the questionnaire at the time of follow-up.

Pregnancy and delivery are recognized as the strongest risk factors for UI in women of reproductive age [2]. Thus, increased awareness among health care providers about promoting continence with PFMT and other modifiable lifestyle factors may be of great importance to reduce UI incidence. Furthermore, identifying women with symptoms in pregnancy or the postnatal period and offering targeted treatment may reduce impact on quality of life and individual suffering from UI. In a committee opinion, the American College of Obstetricians and Gynaecologists proposes a new paradigm for postpartum care including a comprehensive postpartum visit with a full assessment of physical, social and psychological well-being that includes incontinence [23]. In the recent new Australian National Women’s Health Strategy 2020–2030, incontinence was acknowledged as a key health risk for girls and women [24]. Considering that UI in pregnancy and after delivery is associated with an increased risk of UI in the long term, these two recent incentives are most welcome.

## Conclusion

Due to low response rate (35%), we are unable to conclude on the long-term effects of antenatal PFMT on UI. Among the women responding to the 7-year follow-up, we found no group differences in antenatal exercise including PFMT on UI after 7 years. UI in pregnancy was associated with UI in the long term. Regular exercise was not associated with UI. However, incontinent women engaging in regular exercise were more than twice as likely to exercise at lower intensity levels than continent women. Identifying women at risk or experiencing UI during pregnancy and targeted intervention may be important to reduce the risk of UI later in life. The impact of regular exercise and exercise intensity on UI and PFM needs to be examined further.
